# Investigating Differences in Behavior and Brain in Human-Human and Human-Autonomous Vehicle Interactions in Time-Critical Situations

**DOI:** 10.3389/fnrgo.2022.836518

**Published:** 2022-02-24

**Authors:** Anirudh Unni, Alexander Trende, Claire Pauley, Lars Weber, Bianca Biebl, Severin Kacianka, Andreas Lüdtke, Klaus Bengler, Alexander Pretschner, Martin Fränzle, Jochem W. Rieger

**Affiliations:** ^1^Department of Psychology, University of Oldenburg, Oldenburg, Germany; ^2^OFFIS Institute for Information Technology, Division of Transportation Research, Oldenburg, Germany; ^3^Chair of Ergonomics, Technical University of Munich, Garching, Germany; ^4^Chair of Software and Systems Engineering, Technical University of Munich, Garching, Germany; ^5^Department of Computer Science, University of Oldenburg, Oldenburg, Germany

**Keywords:** human-autonomous vehicle interaction, whole-head fNIRS, multivariate logistic ridge regression, valuation of actions, decision-making

## Abstract

Some studies provide evidence that humans could actively exploit the alleged technological advantages of autonomous vehicles (AVs). This implies that humans may tend to interact differently with AVs as compared to human driven vehicles (HVs) with the knowledge that AVs are programmed to be risk-averse. Hence, it is important to investigate how humans interact with AVs in complex traffic situations. Here, we investigated whether participants would value interactions with AVs differently compared to HVs, and if these differences can be characterized on the behavioral and brain-level. We presented participants with a cover story while recording whole-head brain activity using fNIRS that they were driving under time pressure through urban traffic in the presence of other HVs and AVs. Moreover, the AVs were programmed defensively to avoid collisions and had faster braking reaction times than HVs. Participants would receive a monetary reward if they managed to finish the driving block within a given time-limit without risky driving maneuvers. During the drive, participants were repeatedly confronted with left-lane turning situations at unsignalized intersections. They had to stop and find a gap to turn in front of an oncoming stream of vehicles consisting of HVs and AVs. While the behavioral results did not show any significant difference between the safety margin used during the turning maneuvers with respect to AVs or HVs, participants tended to be more certain in their decision-making process while turning in front of AVs as reflected by the smaller variance in the gap size acceptance as compared to HVs. Importantly, using a multivariate logistic regression approach, we were able to predict whether the participants decided to turn in front of HVs or AVs from whole-head fNIRS in the decision-making phase for every participant (mean accuracy = 67.2%, SD = 5%). Channel-wise univariate fNIRS analysis revealed increased brain activation differences for turning in front of AVs compared to HVs in brain areas that represent the valuation of actions taken during decision-making. The insights provided here may be useful for the development of control systems to assess interactions in future mixed traffic environments involving AVs and HVs.

## Introduction

A majority of vehicle accidents are caused by human errors (Singh, 2018). A long-held belief is that the introduction of autonomous vehicles (AVs) in driving will reduce human errors, leading to an overall improvement in terms of driving performance and safety for all traffic participants. However, until a time comes when only AVs travel on roads, human driven vehicles (HVs) and AVs will co-exist in traffic environments. In such mixed traffic environments, the interaction between humans and autonomous agents remains extremely important. This is of concern regarding not only the humans using AVs, but also regarding the interaction between HVs and AVs.

A key aspect for a safe and seamless interaction between HVs and AVs is how human's actions are influenced by AVs in mixed traffic environments. In fact, some studies have shown that pedestrians and human drivers could actively exploit the alleged technological advantages of AVs. For example, the pedestrian or the human driver knows that AVs are programmed to be risk-averse and stop immediately if it detects an obstacle in its path. Armed with this knowledge, drivers and pedestrians may act with impunity while interacting with AVs. Several studies have reported a shift in behavior when humans are interacting with autonomous agents compared to other human agents suggesting that humans might evaluate their own actions differently depending on the type of traffic agent involved. For example, Trende et al. (2019) showed that in time-critical situations, drivers had a significantly higher gap acceptance probability for turning in front of an AV as compared to HV. Moreover, Millard-Ball (2018) showed that pedestrians took advantage of a mildly-mannered AV knowing that the AV will yield at crosswalks, and they can hence cross the road with impunity. Similar results were reported by Liu et al. (2020) where drivers revealed greater intentions to drive aggressively while interacting with AVs as compared to HVs. Such actions of the driver could be constituted as “*misuse of automation,”* a term coined by Parasuraman and Riley (1997). One such type of automation misuse potentially leading to dangerous situations when interacting with AVs is an “*overreliance”* on the automation system (Parasuraman and Manzey, 2010). Overreliance occurs when a driver tends to rely uncritically on the automation without recognizing its limitations or fails to monitor the automation system's behavior (Saffarian et al., 2012; Cunningham and Regan, 2015).

The assessment of safety-critical situations in complex traffic requires significant cognitive resources to form a mental representation of the situation, to identify potentially critical interaction partners and to predict their behavior. The correct estimation and expectation of other's behavior plays a crucial role for safe interaction. In situations where the HV and AV need to interact directly, the driver may tend to underestimate the reaction time of an AV leading to a risky maneuver. The prediction of the AV's behavior in complex traffic situations is based on the driver's mental model of the AV. Mental models are internal representations of a system concerning its characteristics, potentials and limitations that are mainly formed by interacting with the system (Kurpiers et al., 2020). Such mental models can influence information processing, valuation of actions and the resulting decision to act in human-autonomous vehicle interactions. However, it is hard to evaluate such mental models due to their implicit nature and more objective measures are required.

Neurophysiological measurements allow for an objective tracking of cognitive processes such as decision-making. Spatially resolved brain activation measures can be more specific to decision-making processes as they are recorded at the location where these cognitive processes are manifested. This allows us to unravel what goes on in a driver's brain while performing decision-making interactions with technical systems such as AV. Until now, a solid number of neuroimaging studies have been conducted that revealed human brain areas involved in decision-making and characterized their responses in game theoretic frameworks. Much progress has been made in defining game-theoretic building blocks of human decision-making models and implementing these blocks in executable cognitive architectures such as ACT-R (Taatgen et al., 2005). Moreover, neurophysiological research has revealed neural correlates for action-based value signals for reward related decision-making tasks. Some of these brain areas include the prefrontal cortices such as the dorsolateral prefrontal cortex (dlPFC), ventromedial prefrontal cortex (vmPFC), frontal cingulate, anterior orbito- and mediofrontal cortices (Sanfey, 2007; Lee, 2008; Rangel et al., 2008; Gläscher et al., 2009; Ruff and Fehr, 2014). However, very few studies in this field have actually attempted to predict human decision-making interactions from brain activation in realistic situations. Hollmann et al. (2011) employed real-time functional MRI to predict online decisions during social interactions in the ultimatum game from brain activation and to reveal brain areas that signal whether offers were subjectively perceived as unfair. These approaches have been extended from relatively simple operant conditioning in laboratory environments (Schultz, 2002) to decision-making in social context (Sanfey et al., 2003; Sanfey, 2007). However, to the best of our knowledge, no neurophysiological study has compared how interactions with other humans or technical systems such as AVs are reflected in characterizing neural correlates for decision-making in realistic scenarios such as driving using fNIRS.

In this study, we use turning at an intersection as a safety-critical traffic situation, where the driver must directly interact with other traffic participants. Previous studies have reported that between 30 and 40% of crashes are located at or near intersections even though these situations represent only a small percentage of the entire road infrastructure (Tay and Rifaat, 2007; Choi, 2010; Gerstenberger, 2015). A lacking consideration for other road users is the primary reason for accidents when turning according to a report by the German Federal Highway Research Institute (BASt) on intersection-related crash factors (Vollrath et al., 2006; Biebl and Bengler, 2021). When a vehicle stops at an intersection, the driver must observe the oncoming traffic stream before accepting a gap and turning into the desired lane. The gap acceptance problem is one of the main causes for stop-controlled intersection accidents (Yan et al., 2007). Several studies have conducted field observations or driving simulator studies to investigate gap acceptance in these situations (Ragland et al., 2006; Yan et al., 2007; Lord-Attivor and Jha, 2012), leading some of them to predict gap acceptance using statistical models. Lord-Attivor and Jha (2012) collected data from Nigerian intersections and proposed a binary logit model to model gap acceptance behavior. Furthermore, Ragland et al. (2006) analyzed video recordings of five intersections to determine gap acceptance statistics and proposed a logit model predicting gap acceptance probability. Such models can help to design and develop driving assistance decision support systems, which can potentially reduce the number of traffic accidents at the intersections.

The objective of this study is to investigate if there is a difference between a human driver's valuation of actions when an interaction involves technical systems such as AVs as compared to similar interactions with other human beings. In a second step, this paper aims to examine whether these potential differences in human-human and human-autonomous vehicle interactions can be characterized from behavior and neurophysiological whole-head fNIRS brain activation measurements. For this purpose, we conducted an fNIRS-driving simulator study. We measured whole-head brain activation using high density fNIRS throughout the entire driving time to identify neural correlates associated with the valuation of actions during decision-making in the turning situations in human-human and human-autonomous vehicle interactions. We presented the participants with a cover story that they were driving under time pressure through urban traffic in the presence of other HVs and AVs, that the AVs were programmed defensively to avoid collisions and that they had faster braking reaction times than HVs. Participants would receive a monetary reward if they managed to finish the driving block by avoiding risky driving maneuvers within a given time limit. The participants were repeatedly confronted with a left-lane turning situation at unsignalized intersections where they had to decide to turn in front of a HV or an AV. We hypothesize that under time pressure, there is more considerate behavior while interacting with HVs than with AVs as for the latter, there is no safety-critical consequence of one's own actions due to the driver's expectation that AVs drive more cautiously making them more predictable in their driving behavior as compared to HVs. This would be reflected in reduced safety margins (e.g., gap sizes) and increased certainty during the decision-making process while interacting with AVs as compared to HVs. Based on previous research (Sanfey, 2007; Rangel et al., 2008; Ruff and Fehr, 2014), we hypothesize that human-autonomous vehicle interactions cause increased activation modulations in the prefrontal areas such as the dorsolateral and ventrolateral prefrontal, ventromedial prefrontal, frontal midline brain areas and the anterior cingulate cortex, since these brain areas are thought to represent the consideration of values of actions taken during decision-making.

## Materials and Methods

### Participants

Thirteen volunteers (7 females) aged 21–29 years (Mean ± SD = 23.8 ± 2.61) participated in the study. The participants had a mean driving experience of 5.8 years (SD = 2.5). All participants possessed a valid German driving license and gave written informed consent to participate prior to the experiment in accordance with the Declaration of Helsinki. The Ethics Committee of the Carl von Ossietzky University, Oldenburg approved the experimental procedure. Participants received a financial reimbursement of 10 € per hour.

### Experimental Set-Up

**Figure 1:**
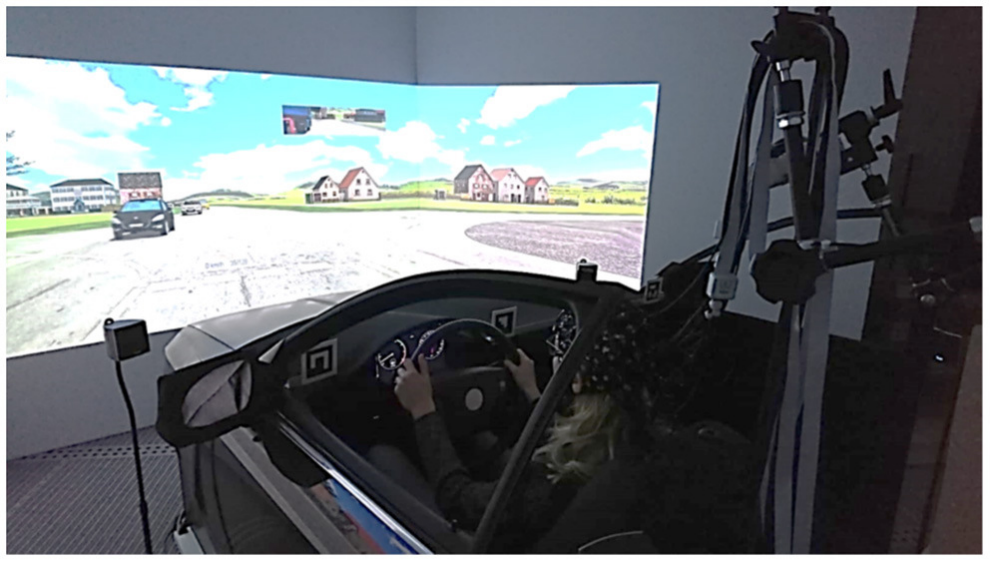
Virtual reality lab driving simulator at OFFIS Institute of Information Technology, Oldenburg—photograph of experimental setup. The participant's brain activity is measured with whole-head fNIRS system while they are driving in the urban traffic.

The experiment was performed in a full-scale fixed-base driving simulator, which offered a 150° field of view (Figure 1). The driving simulator contained a realistic vehicle mock-up. The driving simulator software SILAB (Krueger et al., 2005) was used to simulate the driving scenario. The participants controlled the mock-up car in the driving simulation via a standard interface consisting of a throttle, brake pedal and steering wheel. Behavioral data, such as acceleration, velocity and steering wheel angle were recorded via SILAB.

**Figure 2:**
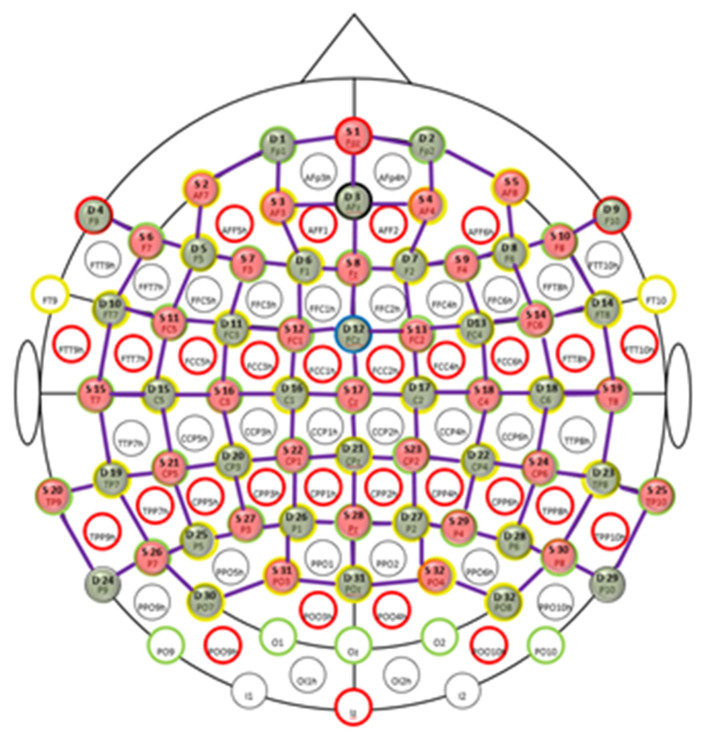
FNIRS probe placement. Topologic layout of the emitters (red disks), detectors (green disks) and the fNIRS channels (purple lines) on a standard 10–20 EEG system. Figure reproduced from NIRStar 15.0 data acquisition software with permission from NIRx Medical Technologies, USA.

Participants' brain activation was measured using a high density, whole-head fNIRS system throughout the entire driving time. fNIRS uses the principle of neurovascular coupling where the neuronal activity is linked to related absorption changes in the sub-surface tissues in localized cerebral blood flow by measuring local concentration changes of oxyhaemoglobin (HbO) and deoxyhaemoglobin (HbR) as correlates of functional brain activity using the modified Beer-Lambert law (Villringer et al., 1993; Sassaroli and Fantini, 2004). We used the NIRScout Extended system (NIRx Medical Technologies) to acquire fNIRS data. The system uses two wavelengths of 760 nm and 850 nm and outputs relative concentration changes of HbO and HbR. Thirty-two optical emitters and detectors were used to obtain close to whole-head coverage. In total, 107 channels (combinations of emitters and receivers) were used to acquire fNIRS data at a sampling frequency of 1.955 Hz (Figure 2). The average distance between an emitter and detector was ~3.5 cm.

Both the fNIRS and driving simulator data were trigger-synchronized during the driving task.

### Experimental Design

**Figure 3:**
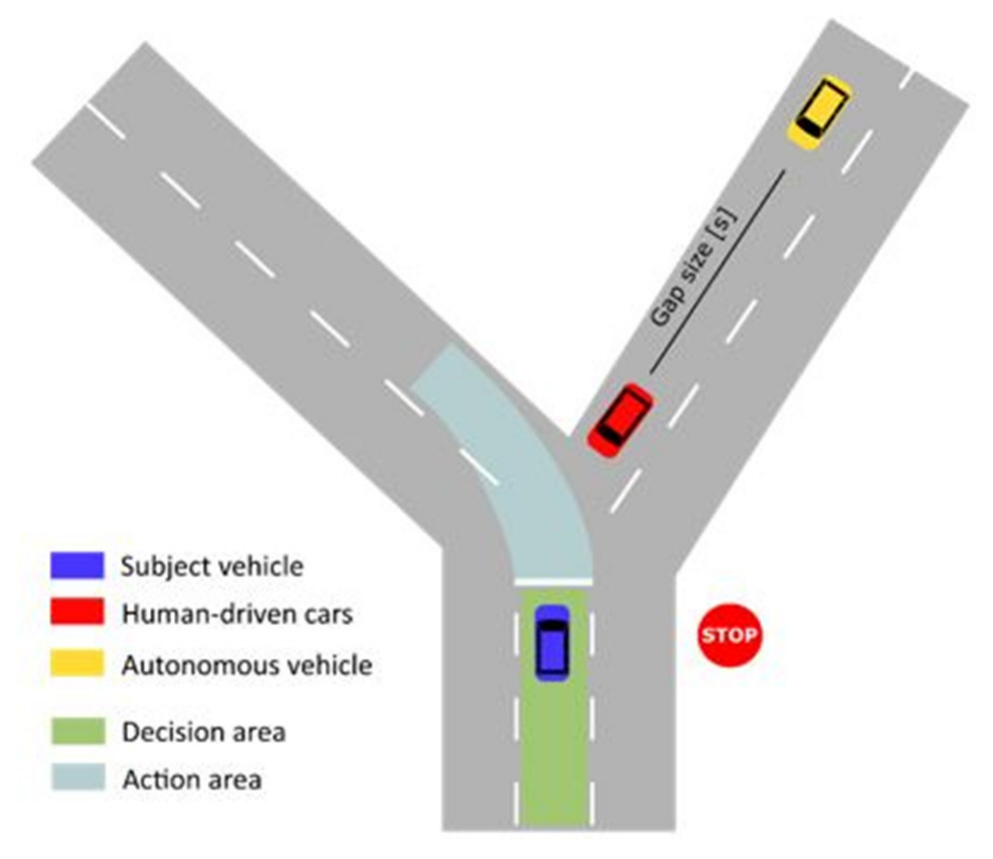
Sketch of intersection. The gap size between two oncoming vehicles defined as the time that passes after the first oncoming vehicle has crossed the intersection until the second vehicle has crossed the intersection.

The driving simulation featured multiple left-turn maneuvers with oncoming traffic in an urban environment. The oncoming vehicles drove at a speed of 50.4 km/h (equivalent to 14 m/s), the speed limit for most urban roads in Germany. Due to a STOP sign, the subject vehicle had to stop at the intersection before accepting a gap and turning into the desired lane (Figure 3). A “gap” represents the opportunity to turn left before an oncoming vehicle. Every gap has an associated gap size that represents the time in seconds that passes after the first of two successive, oncoming vehicles passes the intersection until the second vehicle passed the intersection. The driver faces a series of gaps of different sizes while waiting at the intersection and has the choice to either accept or reject a given gap. Accepting a gap means that the driver completes a left-turn maneuver.

The lane of oncoming traffic was bent slightly to the right (Figure 3). This makes the estimation of the gap sizes between oncoming vehicles easier. The simulated traffic consisted of human driven vehicles (HVs) and autonomous vehicles (AVs) (Trende et al., 2019). The AVs were always yellow cars without a virtual human model visible inside the car. The HVs were represented by cars of other colors (except yellow) where the virtual human model was clearly visible. Before the experiment, the participants were instructed how the AVs will look in the simulation. The participants were told that the AVs are programmed to use a defensive, risk-avoiding driving style (Millard-Ball, 2018). However, in reality, both HV and AV followed the same driving behavior. The number of AVs in the simulation was lower than the number of HVs since automated driving is a novel technique and only a few AVs are available on the market. AVs represented 15% of the simulated cars. While waiting at the intersection in front of the STOP sign, between eight and 10 cars approached the intersection.

We followed Ragland et al. (2006) to design realistic traffic situations. The authors used video data from five intersections in the USA to find the distributions of gaps smaller than 12 s between subsequent cars at intersections. They found that most gaps were 4 s or shorter with the most frequent gap being 2 s. Overall, the gap size distribution could be modeled as a lognormal distribution. We designed the distribution of gaps in our study according to these findings. We decided to present gap sizes between 1 and 6 s during the experiment. As suggested in Yan et al. (2007), we designed the traffic in such a way that the first oncoming vehicles have lower gap sizes. This helps to find minimal gap size acceptances for participants and assures that a suitable gap size for each participant's preferences was presented. We split the oncoming traffic in two groups: The first 4–6 cars have a gap size from a range of 1–3 s. A larger gap in the range of 3.5–6 s was placed after fifth to tenth car, respectively. No vehicles appeared after the tenth car. The sequence of the type of involved traffic agent (i.e., HV or AV) among the stream of oncoming traffic encountered at the intersection varied during one experiment but remained the same for all participants. We performed a training session before the experiment in which the participants drove a short scenario consisting of rural roads and 11 intersections, which took around 10 min. The purpose of the training scenario was to get accustomed to the virtual reality environment and simulator dynamics.

In the experimental session, participants drove 100 intersections consisting of 10 driving blocks with 10 intersections per block. The whole session lasted around 70 min. The participants were asked to stop after 10 intersections and had a break of 1–1.5 min. Time pressure was applied during each block of the experiment. If the participants managed to reach the end of the 10^th^ intersection in a block within 5:30 min, they received a bonus of 1€ per block. The timer and intersection counter were displayed as a Heads-up-Display (HUD) in the simulation. To reach the end of the scenario within the block in the given time limit, the participants had to take at least some of the gaps while waiting at intersections. In principle, participants could have waited until the end of the oncoming stream of vehicles before deciding to turn. However, the time constraints introduced by the bonus discouraged participants to employ such a strategy. Across all participants, only 2 out of the 1,200 turning maneuvers were performed after the last car when the oncoming traffic had already passed.

After the experimental session, the participants were asked to fill out a questionnaire with 4 qualitative questions about trust in AV. The participants choose a score between 1 and 6 for the quantitative items. They rated 4 items related to trust in AV: “I accept AVs on the roads”; “AVs are safer than HVs”; “I trust AVs more than HVs” and “I behaved differently in my interactions with AVs compared to HVs.”

### Data Analysis

The data analysis section consists of three parts: analysis of the driving behavior, analysis of the neurophysiological data and analysis of the questionnaire.

### Behavior Parameters

As presented in other studies (Fitzpatrick, 1991; Ragland et al., 2006), we calculate the gap acceptance probability for each gap size over all participants. The gap acceptance data was extracted based on the positional data of the subject vehicle and oncoming vehicles. We fitted a logistic model to the gap acceptance probability of the participants. The gap acceptance probability was calculated for gap sizes in 0.35 s steps. The logistic model had the following form and two regression parameters to fit.


(1) $$\begin{array}{r} P\left(X,\;m,\;w\right)\;=\;\frac{1}{\left(1\;+\;exp\left(-2\log\left(\frac{1}{0.05}\;-\;1\right)\frac{X\;-\;m}{w}\right)\right)} \end{array}$$

Here, “*X”* represents the gap size, “*m”* is the threshold indicating a 50% gap acceptance and “*w”* is the width describing the difference between 5 and 95% point of the model. *MATLAB 2020* and the *psignifit 4* toolbox (Schütt et al., 2015) were used for fitting the logistic model to the data.

### fNIRS Data Pre-processing

The raw fNIRS data are influenced not only by cortical brain activity but also by other systemic physiological artifacts (cardiac artifacts, respiration rate, and Mayer waves) and movement artifacts causing the signal to be noisy. We pre-processed the raw fNIRS data using the nirsLAB analysis package to reduce the influence of these artifacts (Xu et al., 2014). First, a “*coefficient of variation”* (CV) was computed which is a measure for the signal-to-noise ratio (SNR) from the unfiltered raw data. The CV was calculated as the ratio between the standard deviation and the mean of each NIRS channel over the entire duration of the experiment (Schmitz et al., 2005; Schneider et al., 2011). All channels with a CV >20% were excluded from further analysis. Moreover, we performed band pass filtering of the raw fNIRS data with a high cut-off frequency at 0.1 Hz to attenuate the effects of the above-mentioned physiological artifacts and instrument noise and a low cut-off frequency of 0.01 Hz to reduce the effects of very low frequency and gradual drift in the fNIRS data. Additionally, we visually inspected all channels and deleted those, which were excessively noisy with various spikes. Using these methods, on average, 99 fNIRS channels per participant were included in the subsequent analysis (SD = 8.7). Further, the modified Beer-Lambert's law was applied to convert the raw data from voltage (μ*V*) to relative concentration change (*mmol/l*) (Sassaroli and Fantini, 2004).

The following fNIRS analysis was based on HbR signal, as HbR signals are considered to be less influenced by systemic physiological artifacts like cardiac pulsation, respiration, or Mayer wave fluctuations than HbO (Obrig et al., 2000; Zhang et al., 2005, 2009; Huppert et al., 2009; Suzuki, 2017). Moreover, other studies reported that HbR tends to correlate stronger with the blood oxygenated level dependent (BOLD) response than HbO (MacIntosh et al., 2003; Huppert et al., 2006; Schroeter et al., 2006; Foy et al., 2016).

We performed two types of analyses in order to better understand the neurophysiological activation differences as an index for differences in decision-making while turning in front of HV or AV and to characterize the contribution of these differences on a functional brain-level. The first type was a multivariate decoding modeling framework where our goal was to decode from the whole-head fNIRS activity whether the participant currently decided to turn in front of an HV or AV. The decision-making phase was defined as the decision to turn either in front of a HV or an AV along with the action to execute the decision. This phase corresponded to the timing 2 s before pressing the accelerator and initiating the decision to turn up to 2 s after beginning the turning maneuver for each trial. We selected this 4 s interval for the decision-making phase to account for the hemodynamic delay in the BOLD response measured by fNIRS. In the second type of analysis, we investigated the contribution of the brain activation features to such a decoding model that predicts human-human (turning in front of HVs) or human-autonomous (turning in front of AVs) interactions in the decision-making phase in a group-level by reporting the effect sizes for each fNIRS channel. The following sections provide further details about the methods to implement these analyses.

### Multivariate Cross-Validated Prediction of Turning in Front of HV or AV

The goal of this analysis was to predict whether the participant decided to turn in front of a HV or AV from the pre-processed z-score normalized fNIRS data. First, since there were always more HV trials than AV trials, we balanced the trials by randomly selecting a sample of HV trials matching the number of AV trials available for each participant. Each timepoint (sampling frequency 1.955 Hz) in the 4 s time window during the decision-making phase while turning in front of a HV or an AV was considered as a single sample for the following classification.

The normalized fNIRS data was separated into train and test data. We calculated a multivariate binary logistic ridge regression model implemented in the Glmnet toolbox (Qian et al., 2013) within a 5-fold nested cross-validation on the samples to predict whether a particular timepoint in the fNIRS test data corresponded to human-human or human-autonomous interaction. The optimization of the hyperparameters (number of principal components (PCs) and regularization parameter λ) of the model was carried out in the training phase of the inner cross-validation loop. The outer cross-validation loop tested the generalization the logistic ridge regression model with the optimized hyperparameters to new data. This approach avoids overfitting of the model to the data and provides an estimate of how well the chosen decoding model would predict data that has not been seen previously by the model; for instance, in an online analysis (Hastie et al., 2009; Reichert et al., 2014).

The λ parameter, which determines the overall intensity of regularization of the logistic ridge regression model, was optimized by Glmnet using the training data within the cross-validation (Qian et al., 2013). We first performed a principal component analysis (PCA) on the training set. In this way, the fNIRS training data was transformed into a set of linearly uncorrelated variables called principal components (PCs). By this method, the first PC accounted for the largest variance in the data, and each successive component had the largest possible variance while maintaining orthogonality to the preceding components. The first PC has been shown to be linked to motion artifacts (Brigadoi et al., 2014), and was removed from further analysis. To increase the signal-to-noise ratio (SNR) and limit further analyses to the data explaining the most possible variance, all PCs with eigen values <0.7 were removed as recommended by Jolliffe (1972) on the Kaiser's rule (Kaiser, 1958). This resulted in an average of 13 PCs (SD = 2.4) per participant. The PCA eigen vectors of the training set was used to transform the test dataset in PC space.

Since the output of logistic regression can be interpreted as a class probability, all samples with a model output of *p* ≥ *0.5* were assigned to the class “AV.” This allowed us to calculate the rates at which the model correctly classified the two conditions. In this study, we report model accuracy, which indicates the proportion of correctly classified samples as either turning in front of an AV or a HV. The model accuracy was calculated as follows:


(2) $$\begin{array}{r} Accuracy\left(\%\right)\;=\;\frac{TP_{AV}\;+\;TP_{HV}}{TP_{AV}\;+\;TP_{HV}\;+\;FP_{AV}\;+\;FP_{HV}}*100 \end{array}$$

Here, TP refers to the number of true positives (number of samples correctly classified) and FP refers to the false positives (number of samples incorrectly classified) for the two conditions AV and HV. Further, we also calculated the F1-score, which is a combined harmonic average of the precision and recall measures of the model. The F1-score for AV condition was calculated as follows:


(3) $$\begin{array}{r} F1\text{-}score\;=\;\frac{2*TP_{AV}}{2*TP_{AV}\;+\;FP_{AV}\;+\;FP_{HV}} \end{array}$$

The F1-score for HV condition was also calculated accordingly. We report the final mean model accuracy and F1-score for all participants.

### Characterization of Brain Areas Predictive to Decision-Making Phase in Human-Human and Human-Autonomous Interactions

We aimed to characterize the separability of human-human or human-autonomous vehicle interactions from the channel-wise brain activation features used in the above described multivariate logistic ridge regression model. For this, we performed a channel-wise paired *t-test* from the preprocessed fNIRS data for the two conditions AV and HV on a single-subject level. To generalize the individual t-statistics brain maps to our test sample, the channel-wise single-subject *t-statistics* (*t*) were weighted with the participant's average model accuracy from the multivariate logistic ridge regression (*Accuracy*_*mvr*_) to compute a weighted average t-statistics (*t*_*avg*_) across the test sample (Unni et al., 2017) as shown below.


(4) $$\begin{array}{r} t_{avg}\left(i\right)\;=\;\frac{\sum_{i,\;n\;=\;1}^{i,\;n}t\left(i\right)*\;Accuracy_{mvr}\left(n\right)}{\sum_{1}^{n}Accuracy_{mvr}\left(n\right)} \end{array}$$

Here, *i* refers to the total number of fNIRS channels and *n* indicates the total number of participants. We calculated *Cohen's d* for each channel from *t*_*avg*_ to indicate the effect sizes in sensor space.


(5) $$\begin{array}{r} Cohen^{\prime }s\;d\left(i\right)\;=\;\frac{{\left(t_{avg}\left(i\right)\right)}^{2}}{\sqrt{df}} \end{array}$$

Here, df refers to the degrees of freedom. We report these *Cohen's d* brain maps for the group-level analyses.

## Results

Data from one participant was excluded due to simulator sickness during the experiment. Thus, data from 12 participants are reported in the following sections.

### Questionnaire Results

The results of the trust-related items from the questionnaire are shown in Table 1. The mean score for the overall trust-related items in the questionnaire was 3.8 (out of 6) indicating a high trust in AVs. The Cronbach alpha for the trust-related items was 0.78, indicating that these items have an acceptable reliability or internal consistency. Furthermore, 7 out of 12 participants stated that they turned in front of an AV preferably, when asked about a specific strategy for AVs. It is possible that these participants may feel that the interaction with a programmed vehicle is more controllable than with a human driver due to the AVs' perceived predictability. Another possible explanation could be that the participants potentially tried to exploit the defensive programming behavior and driving performance of AVs solely based on the cover story to gain a temporal advantage during the drive and achieve the bonus.

**Table 1:** Results from the trust-related items of the post-experiment questionnaire.

**Item**	**Mean score ±SD**
I accept AVs on the roads.	4.2 ± 1.3
AVs are safer than HVs.	4.0 ± 1.5
I trust AVs more than HVs.	3.9 ± 1.4
I behaved differently in my interactions with AVs compared to HVs.	3.2 ± 1.2
Overall	3.8 ± 0.9

### Behavioral Results

**Figure 4:**
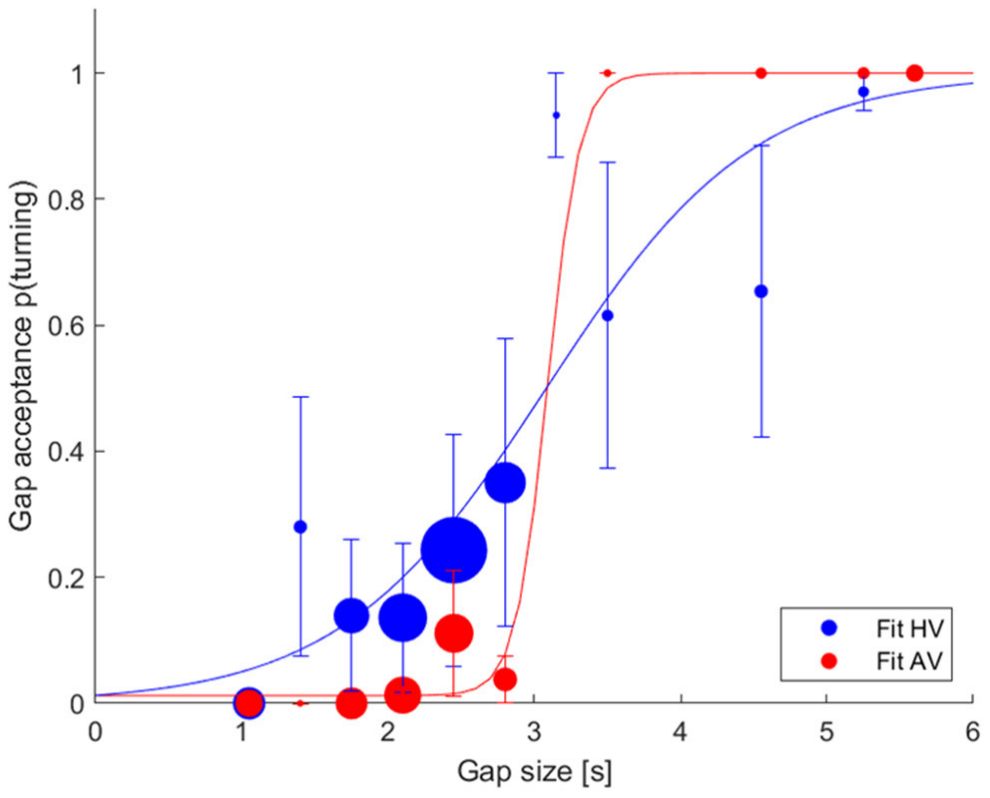
Gap acceptance vs. gap size over all participants for HV (blue) and AV (red). The marker size represents the total number of events for the corresponding gap size. A logistic model was fit to each condition.

Figure 4 shows the gap acceptance probability in relation to the gap sizes by fitting the logistic model for HVs and AVs. Gap sizes were grouped in 0.35 second steps. The gap acceptance events were pooled over all participants. The models' widths (w) describing the difference between the 5 and 95% point of the model for AVs and HVs were w_AV_ = 0.65 s (0.59–1.25 s) and w_HV_ = 4.17 s (2.87–5.04 s), respectively. This is indicated by a steeper slope for AV as compared to HV in Figure 4.

The models' threshold values indicating the 50% gap acceptance for AVs and HVs were m_AV_ = 3.09 s (2.96–3.20 s) and m_HV_ = 3.08 s (2.75–3.30 s), respectively. The difference in the threshold values is 0.04 m. The models' threshold values and their corresponding confidence intervals show an overlap, suggesting that these distributions do not differ significantly.

### Prediction of Human-Human or Human-Autonomous Interaction From the Decoding Model

Using the multivariate logistic ridge regression model, we were able to predict the type of traffic agent (AV or HV) in the decision-making phase from whole-head fNIRS brain activation measurements with an average prediction accuracy and F1-score of 67.2% (SD = 3%) and 0.67 (SD = 0.05) respectively, across all participants. Prediction accuracies obtained in line with the measured dataset exceeded the 95% confidence interval (CI) for guessing for all participants. Table 2 reports the individual prediction results for all participants along with the CI for the empirical chance level. All multivariate predictions reported in Table 2 were determined on a 5-fold cross-validation to evaluate the model's generalization to new data to approximate an online analysis.

**Table 2:** Five-fold cross-validated predictions of AV and HV from HbR fNIRS measurements using multivariate logistic ridge regression analysis for all participants.

**Participant**	**1**	**2**	**4**	**5**	**6**	**7**	**8**	**9**	**10**	**11**	**12**	**13**	**Mean**
Accuracy in % (SD)	69.9 (0.04)	56.8 (0.04)	67.9 (0.03)	68.5 (0.03)	59.5 (0.05)	68.1 (0.04)	65.4 (0.03)	68.7 (0.04)	66.0 (0.04)	66.3 (0.04)	75.9 (0.04)	73.7 (0.05)	67.2 (0.05)
F1-score (SD)	0.70 (0.04)	0.57 (0.02)	0.67 (0.03)	0.69 (0.03)	0.61 (0.05)	0.69 (0.04)	0.65 (0.03)	0.69 (0.04)	0.66 (0.04)	0.64 (0.04)	0.77 (0.04)	0.74 (0.05)	0.67 (0.05)
5–95% accuracy CI for empirical chance level (SD)	45.8–54.1	46.9–53.1	46.2–53.8	46.2–53.5	46.3–54.1	46.0–54.2	46.6–53.4	45.6–54.3	46.0–53.6	46.2–53.9	45.3–54.2	45.6–54.5	46.0–53.9 (0.004)

This is to our knowledge the first evidence that brain processes may differ in the interactions between human driven and autonomous cars. Together with the behavioral results, this suggests that human driver may assess the interactions with AV differently from interactions with HV.

### Effect Sizes Discriminating Turning in Front of AV vs. HV From fNIRS Brain Activation

**Figure 5:**
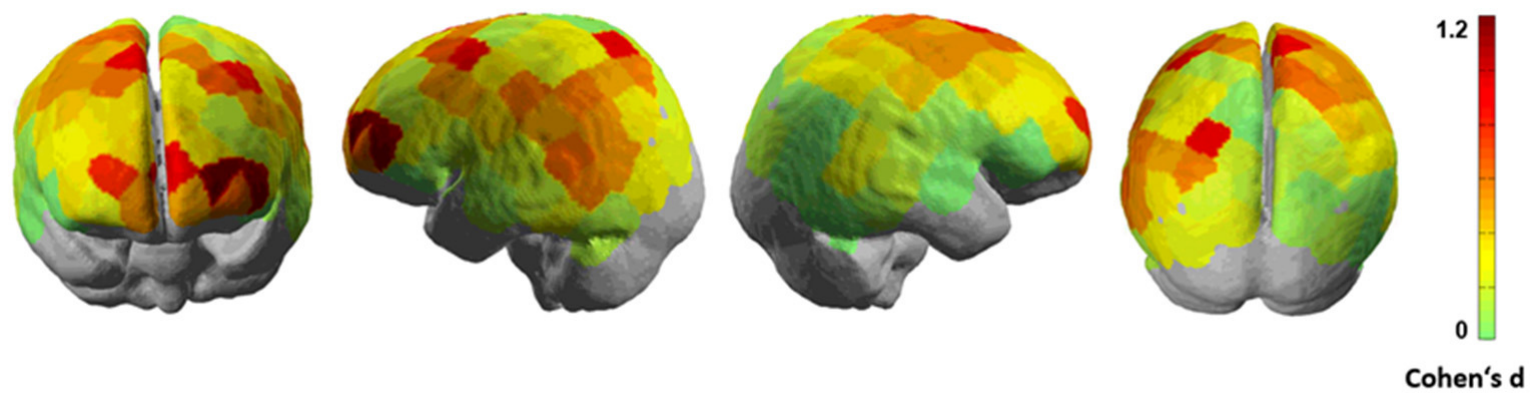
Cohen's d brain maps representing effect sizes computed from channel-wise weighted averaged t-statistics (t_avg_) for the group-level analysis. Moderate to high Cohen's d values (0.8–1.2) show medium to large effect sizes indicating increased activation differences for the decision-making phase during turning in front of AV as compared to HV.

Figure 5 shows the Cohen's d brain maps for the group-level analysis. We visualized the averaged brain map on the MNI 152 brain in Neurosynth^1^ and used MRIcron^2^ to determine MNI coordinates and the corresponding Brodmann areas (BA) for the brain areas with increased activation differences during the left-lane turning decision-making phases for AVs as compared to HVs.

Table 3 lists the brain areas, the MNI-coordinates of the difference maxima and the Cohen's d values as indicators of the effect sizes from the group-level analyses (*n* = 12).

**Table 3:** Brain areas showing increased activation differences in the decision-making phase during turning in front of AVs compared to HVs.

**Brain areas**	**Putative Brodmann area (BA)**	**X**	**Y**	**Z**	**Cohen's d**
Left dorsolateral prefrontal	46	−26	62	26	1.20
Right dorsolateral prefrontal	46	24	60	32	0.81
Ventromedial prefrontal	10	4	58	28	0.93
Left ventrolateral prefrontal	45	−48	42	28	1.10
Left superior frontal gyrus	6	−34	10	60	0.87
Right superior frontal gyrus	6	16	4	76	0.93
Left superior parietal	7	−28	−62	68	0.86

*The approximate MNI coordinates of activation differences along with the putative Brodmann areas and their Cohen's d values are shown*.

The results showed the largest effect sizes of brain activation in the prefrontal cortex (PFC), reflecting activation changes in the left and right dorsolateral areas (dlPFC; putative BA 46) and the left ventrolateral prefrontal (vlPFC; putative BA 45) areas (Cohen's d ~ 0.9–1.2). Additionally, the ventromedial prefrontal areas (vmPFC; putative BA 10) also indicate increased activation differences while turning in front of AV as compared to HV (Cohen's d ~ 0.9). These prefrontal areas have been previously implicated in the valuation of actions during decision-making (Sanfey, 2007; Lee, 2008; Rangel et al., 2008; Hollmann et al., 2011; Ruff and Fehr, 2014). Furthermore, the superior frontal gyrus (SFG) and parts of the motor cortices (putative BA 6) also show increased activation differences between the turning phases of AV and HV. Moreover, some informative channels can be seen in the left superior parietal areas (putative BA 7). Overall, our results demonstrate a consistent difference in activation at the brain-level and these activation differences occur in brain areas that have been previously related to decision-making.

## Discussion

The main promise of autonomous driving is that AVs will reduce traffic accidents caused by human errors and hence be safer than HVs. The aim of this study was to investigate if there is a difference between the valuation of actions when an interaction involves technical systems such as AVs as compared to similar interactions with other HVs. Moreover, we wanted to investigate if these potential differences in human-human and human-autonomous vehicle interactions can be characterized from behavior and neurophysiological whole-head fNIRS brain activation measurements. We believe that this research goal is extremely relevant in the present situation since some studies have shown that humans could actively exploit the predictable and safe behavior of AVs. With the knowledge that AVs are programmed to be risk-averse, humans tend to act with impunity while interacting with AVs (Millard-Ball, 2018; Trende et al., 2019; Liu et al., 2020). Our results provide evidence that humans show a difference in the valuation of actions in the decisions they make in such situations depending on whether they interact with an AV or a HV and this is expressed in fNIRS brain activation and partly in the behavioral tendencies.

We investigated differences in human-human and human-autonomous interactions using a full-scale fixed base driving. In our cover story, we mentioned that the AVs were defensively programmed in an interaction and drove conservatively to avoid collisions and had faster braking reaction times than HVs as this is the expected programming of AVs (Zhan et al., 2016; Li and Sun, 2018). However, both, AVs and HVs were simulated according to the same driving behavior.

Results of our gap acceptance model showed that the confidence intervals of the threshold parameter (m) of the gap acceptance models overlapped, indicating that there is no significant difference between the safety margin used during the turning maneuvers with respect to AVs or HVs. Furthermore, we observed differences in the model widths, which describes the 5–95% point of the AV and HV models. The models' width parameters indicated that the AV distribution is steeper than the HV distribution. The steeper slope for AV could be interpreted as participants tended to be more certain in their decision-making process while turning in front of AV as reflected by the smaller variance in gap size acceptance as compared to HV. Similarly, the shallower slope for HV could indicate that participants have larger uncertainty in their decision-making process while interacting with HV during the lane-turning maneuver. We assume that the participants may have felt more certain while interacting with AVs due to their perceived predictability and potentially tried to exploit the defensive strategy of AVs. The participants may have overestimated the AVs' alleged defensive behavior despite the fact that AVs showed the same driving behavior as HVs in order to gain a temporal advantage in the experiment. This assumption is further supported by the results of a trust questionnaire. The mean score for the item “I trust AVs more than HVs” was 3.9 (on a scale of 1–6) supporting the claim that the participants may have overestimated the AVs' alleged driving behavior and underestimated the technological limitations of AVs. This automation complacency regarding the AVs' safe functioning in the simulation could potentially lead to dangerous situations (Parasuraman and Riley, 1997).

The neurophysiological results indicate that our approach of using whole-head fNIRS in combination with a cross-validated multivariate logistic ridge regression is suitable to predict the type of involved traffic agent (AV or HV) while making a decision to turn. This approach allowed us to exploit the spatial specificity of whole-head fNIRS, in order to predict the traffic agent involved at the crossing with an average accuracy of approximately 67% (SD = 3%) across all participants and up to a maximum of almost 76% on a single-subject level. It is important to note that these cross-validated predictions are obtained from just 4 s of fNIRS data in the decision-making phase demonstrating that our approach of combining multivariate logistic ridge regression and cross-validation and exploiting the spatial specificity of whole-head fNIRS has the potential to predict the interaction partner in time-critical situations. While the predictions might not be very high, we have previously shown that even imperfect predictions regarding the driver's intent can be useful to develop driver models which can lead to increased safety during interactions between AVs and HVs in mixed traffic environments (Damm et al., 2019).

To characterize the neural correlates for the decision-making phase in human-human and human-autonomous interactions, we computed the channel-wise Cohen's d measures as effect sizes for the fNIRS brain activation over the AV and HV turning conditions in a group-level analysis. Our initial hypothesis was that human-autonomous vehicle interaction would result in increased modulations in the prefrontal areas such as the dorsal and ventral frontal areas, frontal midline brain areas such as the ventromedial prefrontal areas and the anterior cingulate cortex, since these brain areas are thought to represent the values of actions taken (Sanfey, 2007; Rangel et al., 2008; Ruff and Fehr, 2014). Due to the limited spatial depth of fNIRS, we could not observe the activity in the anterior cingulate cortex. However, the group-level analysis revealed increased fNIRS activation in the prefrontal areas such as the dorsolateral (putative BA 46), left ventrolateral (putative BA 45), ventromedial prefrontal areas (putative BA 10), the superior frontal gyrus and parts of the motor cortices (putative BA 6) when participants turned in front of the AV as compared to HV. The activation in these brain areas could potentially reflect the differences in valuation of actions when turning in front of an AV as compared to HV. The prefrontal cortex is an important brain area that subserves higher order executive functions necessary for the cognitive control of behavior and decision-making. The dorsolateral prefrontal areas (putative BA 46) show increased activation during risky decision-making where costs and benefits are weighed (Duncan et al., 1996). BA 45 has been associated with reasoning and goal-intensive processing (Goel et al., 1998; Fincham et al., 2002). The ventromedial prefrontal cortex (putative BA 10) has been shown to be a part of the reward-processing mechanism elicited by emotional processes, which plays a vital role in determining value-based decision-making (Sanfey, 2007). Moreover, some studies have shown the role of the dorsolateral and ventromedial prefrontal areas to be involved in uncertainty during the decision-making processes (Schienle et al., 2010; Stern et al., 2010; Wever et al., 2015; Tomov et al., 2020). This can be linked to our interpretation of our behavioral results which show a difference in the certainty of the driver during the planning and execution of the turning maneuver in the decision-making phase while interacting with AVs or HVs.

Previous studies have shown the role of the superior frontal gyrus in processing emotions and self-reflections in decision-making (Deppe et al., 2005; Goldberg et al., 2006). Additionally, the involvement of the BA 6 in motor functioning such as planning and execution of motor activities is well-known (Catalan et al., 1998; Hanakawa et al., 2008) suggesting differences in the underlying brain processes during interactions with AVs and HVs.

In our study, some participants (7 out of 12) mentioned that they deliberately took the gap in front of the AVs because they assumed it would brake due to the alleged defensive behavior. This is a dangerous assumption since all the vehicles in the simulation including HVs and AVs were simulated according to the same driving behavior. The participants overestimated the behavior and driving performance of the vehicles solely based on the cover story about the defensive programming of the vehicles and ignored the visual evidence based on the similar driving behavior of AVs and HVs. This is a classic example of “misuse of automation” as defined by Parasuraman and Riley (1997) as an overreliance of automation. This automation complacency may lead to dangerous traffic situations or even accidents in case of excessive overestimation of the reaction time of the AV or sensor failure (Parasuraman and Manzey, 2010). Most of the participants in this study believed that AVs are safer. The findings of this study may be important in mixed traffic environments where both HVs and AVs are participating in the traffic. The software controlling AVs should be able to account for the fact that humans may behave riskier during interactions. Furthermore, it would be interesting to investigate how human drivers would behave if the AVs were able to retaliate uncooperative or risky driving behavior by providing clearly visible cues. Future studies could investigate if the behavior of the human driver changes and if this is reflected in a change of the action valuation signals in the brain activation becoming more similar to the activations observed in interactions with HVs.

The current study has a few limitations. The experimental design of the gap sizes did not feature sufficient samples with gap sizes in the range of 3–6 s. This leads to fewer events within this range. Furthermore, it should be mentioned that the experiment was conducted with a rather homogenous participant pool. The participants were mainly between 21 and 29 years and from an academic background. This group is generally associated to have high trust in technology (Kennedy et al., 2008) which may have an impact on the results from the subjective questionnaire regarding high trust in AV. We suspect that participants with low trust in technology in general and less trust in the safe functioning of AVs in particular will behave differently in such an experiment leading to a shallower slope in gap acceptance function for AVs. In this study, the only relevant factors for the gap acceptance model were the gap size and traffic agent involved, i.e., HVs or AVs. Several studies argue that the gap acceptance also depends on personal characteristics such as age, gender, or intersection characteristics (Darzentas et al., 1980; Bottom and Ashworth, 2007; Yan et al., 2007) which have not been considered in this study.

The brain areas characterized in this study have been shown to be involved in determining the valuation of actions during social interactions in lab-based settings. These neural correlates could be used to develop control systems for interactions with AVs at intersections based on the behavioral tendencies of the driver. Moreover, neurophysiological measures could be used as an indicator to predict the intent of the driver in such human-autonomous interactions. Furthermore, integrating such neurophysiological sensors in control systems could potentially optimize the performance of the AVs under safety constraints in mixed traffic environments in the future.

## Data Availability Statement

The raw data supporting the conclusions of this article will be made available by the authors, without undue reservation.

## Ethics Statement

The studies involving human participants were reviewed and approved by the Ethics Committee of the Carl von Ossietzky University, Oldenburg. The participants provided their written informed consent to participate in this study.

## Author Contributions

AL, KB, AP, MF, and JR planned the research. AT and LW developed the experimental paradigm with support from BB and SK. AU, AT, and CP carried out the data collection and the data analysis. AU and AT prepared the manuscript. All authors provided feedback. All authors contributed to the article and approved the submitted version.

## Funding

This work was supported by the Deutsche Forschungsgemeinschaft (DFG) under grant numbers RI 1511/3-1 to JR, LU 1880/3-1 to AL, and BE 4532/15-1 to KB (Learning from Humans—Building for Humans), PR 1266/3-1 to AP (Design Paradigms for Societal-Scale Cyber-Physical Systems), and FR 2715/4-1 (Integrated Socio-technical Models for Conflict Resolution and Causal Reasoning) to MF.

## Conflict of Interest

The authors declare that the research was conducted in the absence of any commercial or financial relationships that could be construed as a potential conflict of interest.

## Publisher's Note

All claims expressed in this article are solely those of the authors and do not necessarily represent those of their affiliated organizations, or those of the publisher, the editors and the reviewers. Any product that may be evaluated in this article, or claim that may be made by its manufacturer, is not guaranteed or endorsed by the publisher.

## Footnotes

^1^ http://neurosynth.org

^2^ https://www.nitrc.org/projects/mricron
